# Variation by ethnic group in premature mortality risk following self-harm: a multicentre cohort study in England

**DOI:** 10.1186/s12888-015-0637-0

**Published:** 2015-10-19

**Authors:** Pauline Turnbull, Roger Webb, Nav Kapur, Caroline Clements, Helen Bergen, Keith Hawton, Jennifer Ness, Keith Waters, Ellen Townsend, Jayne Cooper

**Affiliations:** 1 Centre for Mental Health and Safety, Centre for Suicide Prevention, The University of Manchester, Manchester, UK; 2 Manchester Mental Health and Social Care Trust, Manchester, UK; 3 Centre for Suicide Research, Department of Psychiatry, Warneford Hospital, The University of Oxford, Oxford, UK; 4 Derbyshire Healthcare NHS Foundation Trust, Royal Derby Hospital, Derby, UK; 5 Self-Harm Research Group, School of Psychology, The University of Nottingham, Nottingham, UK

**Keywords:** Self-harm, Ethnicity, Mortality

## Abstract

**Background:**

Incidence and risk factors for self-harm vary according to ethnicity. People who self-harm have been shown to have increased risk of premature death, but little is known about mortality following self-harm in ethnic minority groups.

**Methods:**

A prospective cohort study of self-harm presentations to three English cities (Derby, Manchester, Oxford) between 2000 and 2010. We linked to a national mortality dataset to investigate premature death in South Asian and Black people in comparison with White people to the end of 2012.

**Results:**

Ethnicity was known for 72 % of the 28,512 study cohort members: 88 % were White, 5 % were South Asian, and 3 % were Black. After adjusting for age, gender and area-level socioeconomic deprivation, the risk of all-cause mortality was lower in South Asian (hazard ratio [HR] 0.51, 95 % confidence interval [CI] 0.42 – 0.62) and Black people (HR 0.46, 95 % CI 0.39 – 0.55) versus White people. Suicide risk was significantly lower in Black people (HR 0.43, 95 % CI 0.19 – 0.97) than in White people. Prevalence of risk factors for premature death, such as previous self-harm, psychiatric treatment or concurrent alcohol misuse, was lower in South Asian and Black people than in White people.

**Conclusions:**

The risk of death following self-harm is lower in South Asian and Black people than White people in the UK, and they also have lower prevalence of risk factors for premature death. Awareness of both protective and risk factors might help to inform clinical decisions following assessment.

## Background

People who self-harm are at increased risk of further suicidal behaviour including suicide [1–3]. A recent study using data from the Multicentre Study of Self-Harm in England found that people who self-harm also have increased risk of premature death from all causes [4]. Death occurred more in men, and those with alcohol and physical health problems. An investigation of suicide and other unnatural death following self-harm identified additional risk factors: being aged older than 35 years, psychiatric treatment, mental health problems, and previous self-harm [5].

Little is known about mortality following self-harm among ethnic minority groups in the UK. However, there is evidence that self-harm repetition is lower amongst South Asian and Black people than White people, although incidence in the younger female ethnic minority groups appears to be at least equivalent to the White population [6–8]. Of South Asian and Black people who self-harm, those who repeat have similar social and clinical characteristics to White patients who repeat self-harm [9].

Ethnicity-related health inequalities may be influenced by genetic or cultural factors, and could reflect differences in access to treatment and rehabilitation, as well as socio-economic status (SES). Almost 45 % of people from ethnic minority groups live in the most deprived fifth of local authorities in England [10]. In fact, a recent investigation into ethnicity and mortality in the UK showed that elevated risk of premature mortality among UK-born Black-Caribbean people was accounted for by individual-level SES and neighbourhood effects [11]. People who present to hospital following self-harm are socially disadvantaged in comparison to the general population in England [12], and so it is likely that White people who self-harm are similar in socio-economic status to South Asian and Black people who self-harm. We hypothesised that South Asian and Black people would have a lower risk of mortality following self-harm than White people in this vulnerable population, due to previously reported lower risk of self-harm repetition in these groups. Previous investigation of the Multicentre Study of Self-Harm data reported that people who self-harm are at increased risk of premature death [4]. Our aim was to use this data to investigate mortality following self-harm in South Asian and Black people in comparison with White people, and to examine the prevalence of characteristics associated with increased risk of premature death in these groups.

## Methods

We conducted a prospective cohort study from 2000 to 2010 in Manchester, Oxford and Derby as part of the Multicentre Study for Self-Harm in England. Data were collected for all non-fatal self-harm presentations to three emergency departments (EDs) in Manchester, one in Oxford and one in Derby (originally two hospitals which merged into one in 2009). An established monitoring system for each centre was used to retrieve data, as has been detailed previously [13]. Self-harm attendances were identified through detailed examination of emergency department records, and were defined consistently across centres as intentional self-poisoning or self-injury, irrespective of type of motivation [14]. Following self-harm, around half of patients received a psychosocial assessment, and clinicians recorded a wide-range of demographic, clinical and hospital-management data, as well as information on problems experienced and alcohol consumption within six hours of harm. For individuals who did not receive a psychosocial assessment, limited information was collected by research clerks from medical records. In Manchester, data were collected on assessed cases only prior to September 2002 and both assessed and non-assessed individuals from then onwards. Ethnicity classification was assigned by the treating clinician at the time of admission according to standard UK national 2001 Census categories, or attributed later using information from the ED patient record system. The three cities have higher proportions of ethnic minority groups than the national average (Manchester 34 %, Derby 20 %, Oxford 22 % versus 15 % for the whole of England) [15]. Ethnic groups were combined into broad categories – South Asian people (Indian, Pakistani and Bangladeshi origin) and Black people (Black African-Caribbean and Black Other), with White people (White British, Irish, or White Other) as the reference group. We did not analyse those of ethnicities in the ‘other’ category, as this was a diverse group with low numbers. A lower age limit of 16 years was applied.

Postcodes of residence were linked to the Index of Multiple Deprivation (IMD) 2007 in England, a measure of relative deprivation according to the following domains: income, employment, health and disability, education, skills and training, barriers to housing and services, living environment, and crime. A higher score indicates greater deprivation [16]. When the 354 local authority areas in England were scored according to the IMD 2007, Manchester was ranked 4^th^ most deprived, Derby 69^th^, and Oxford 155^th^. Deprivation scores of the Census Area Ward in which each patient resided were grouped into tertiles according to the distribution of IMD values across the combined populations of the three centres.

Mortality information was supplied by the Medical Research Information Service (MRIS), more recently the Data Linkage and Extract Service, which traced and flagged individuals with the Central Health Register Inquiry System for patients in England and Wales, and equivalent sources in Scotland. Individuals were traced using name, gender, date of birth, NHS number, and postcode of last address, and were followed up to December 31^st^ 2012 (i.e. a minimum follow up period of two years). Follow-up for individuals ended when they died or emigrated. We report hazard ratios for all causes of death and for death by internal causes (International Statistical Classification of Diseases and Related Health problems 10^th^ Revision [ICD-10] A00 – R99 [17]) and external causes (ICD-10 codes V01 – Y98). Internal causes are natural causes of death, including deaths by cancer, infection, and cardiovascular disease amongst others. External causes include deaths receiving a suicide or open verdict at coroner’s inquest, as well as accidental deaths. Where we have reported hazard ratios for suicide, our case definition included open verdicts as is common in UK suicide research, in recognition that the majority of these deaths are probable suicides [18].

### Statistical analysis

Analyses were carried out using Stata version 12.1 for Windows, and were based on the individuals’ first episode of self-harm recorded during the study period. We calculated Cox proportional hazards models to determine hazard ratios for death by South Asian and Black people in comparison to White people. Both unadjusted and adjusted (aged over 35, sex, and deprivation tertile) models are presented. Differences between South Asian and Black people compared with White people were explored using chi square tests with respect to characteristics associated with increased risk of premature death in previous studies [4, 5]. We also estimated hazard ratios for death among South Asian and Black people combined compared with White people, and present these unadjusted and adjusted for risk factors for premature death.

### Ethical approval

The self-harm monitoring system in Oxford was approved by South Central–Berkshire National Research Ethics Service, and Derbyshire Research Ethics Committee approved the study in Derby. Both were granted approval to obtain data for self-harm in local and multicentre projects. The self-harm monitoring in Manchester was reviewed by South Manchester Research Ethics Committee and was deemed not to require approval as the monitoring is conducted as part of a clinical audit system. All centres are compliant with the provisions of the Data Protection Act of 1998. All centres have approval under Section 251 of the NHS Act 2006 regarding the collection and use of patient-identifiable information and to send patient details to the Data Linkage Service.

## Results

Ethnicity was known for 20,652/28,512 (72 %) of the individuals presenting to the study EDs following a self-harm episode between 2000 and 2010 (median age 30, range 16-97). Of these, 18,255 (88 %) people were White (median age 31, range 16–97), and the remainder were from an ethnic minority, including 1068 (5 %) South Asian (median age 25, range 16-81) and 637 (3 %) Black people (median age 26, range 16-82). The remaining 692 individuals were from other ethnic groups, including mixed race, Chinese, and other Asian origin groups. These people were excluded from the analysis due to their heterogeneous nature when grouped, and small numbers of individual subgroups. Follow-up mortality data were available for 18,190 (99.6 %) White people, 1066 (99.8 %) South Asian people and 634 (99.5 %) Black people. The proportion of people in each ethnic group presenting to hospital following self-harm was consistent over the time period investigated, the total numbers in each group are shown in Table 1. Missing data were excluded from the relevant parts of the analysis.

**Table 1:** Ethnic group of people presenting to hospital following self-harm across the three study cities

	White *N* = 18,255 (%)	South Asian *N* = 1,068 (%)	Black *N* = 637 (%)
City 1	9,984 (89.0)	780 (7.0)	459 (4.1)
City 2	3,676 (94.1)	133 (3.4)	97 (2.5)
City 3	4,595 (95.1)	155 (3.2)	81 (1.7)

### Hazard ratios for mortality

Cox proportional hazards models were fitted for South Asian and Black people in comparison to White people, for all-cause mortality, all internal causes of death, all external causes of death, and suicides (Table 2). South Asian people had significantly lower rates of all-cause, internal, and external causes of death following self-harm than White people, and a lower rate of suicide (though beyond conventional statistical significance: *p* = 0.06). Black people had significantly lower rates of all-cause, internal and external causes of mortality following self-harm than White people; risk of suicide was also significantly lower (*p* = 0.04).

**Table 2:** All-cause and cause-specific unadjusted mortality hazard ratios for South Asian and Black people in comparison with White people

	White	South Asian		Black	
	*N* = 18,255 (%)	*N* = 1,068 (%)	Hazard Ratio (95 % CI)	*N* = 637 (%)	Hazard Ratio (95 % CI)
All causes	1,949 (7.6)	35 (2.7)	0.35 (0.26 – 0.46)***	20 (2.5)	0.33 (0.27 – 0.42)***
Internal causes	1,325 (5.1)	19 (1.5)	0.27 (0.15 – 0.51)***	11 (1.4)	0.27 (0.15 – 0.49)***
External causes^Ϯ^	624 (2.4)	16 (1.3)	0.50 (0.38 – 0.66)***	9 (1.1)	0.47 (0.31 – 0.71)***
Suicide and undetermined	375 (1.5)	10 (0.8)	0.52 (0.26 – 1.02)	5 (0.6)	0.43 (0.19 – 0.97)*

^Ϯ^including suicide and undetermined

*p < 0.05, **p < 0.01, ***p < 0.001

**Table 3:** Prevalence of risk factors for premature death among South Asian and Black people in comparison with White people

	White		South Asian			Black		
	*N* = 25,798	%	*N* = 1,282	%	*p*	*N* = 796	%	*p*
Aged over 35	11,231	44	262	20	<0.01	207	26	<0.01
Male	11,103	43	372	29	<0.01	271	34	<0.01
Unemployed	7,248	32	261	23	<0.01	247	35	0.11
Most deprived tertile*	8,146	34	537	44	<0.01	424	56	<0.01
Precipitating problems:								
Physical health problem	2,253	10	78	7	<0.01	57	9	0.18
Mental health problem	4,536	21	140	13	<0.01	112	17	0.02
Clinical characteristics:								
Alcohol involved at attempt	12,703	59	174	17	<0.01	255	39	<0.01
Any previous self-harm	13,522	62	454	43	<0.01	315	48	<0.01
Current psychiatric treatment	9,185	42	252	24	<0.01	184	28	<0.01

*Index of Multiple Deprivation

**Fig. 1:**
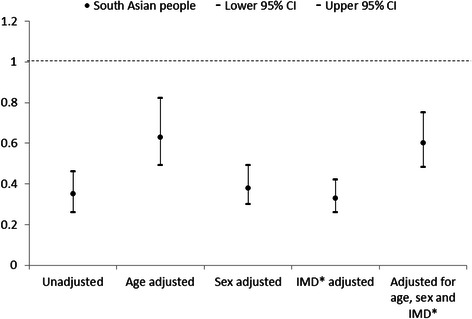
All-cause mortality hazard ratios for South Asian people in comparison with White people adjusted for age, sex, and deprivation tertile. *Index of Multiple Deprivation

**Fig. 2:**
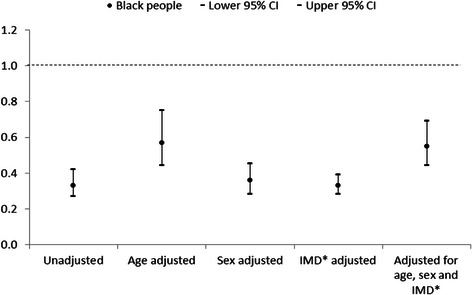
All-cause mortality hazard ratios for Black people in comparison with White people adjusted for age, sex, and deprivation tertile. *Index of Multiple Deprivation

All-cause mortality was adjusted separately for age, sex, and deprivation tertile, then all three factors combined (Figs. 1 and 2). Age showed the greatest confounding effect, with minimal confounding by gender and deprivation. Adjusting for age, sex, and deprivation tertile together attenuated the group differences, altering the unadjusted hazard ratio for South Asians from 0.35 (95 % CI 0.26 – 0.46) to 0.51 (95 % CI 0.42 – 0.62), and the unadjusted hazard ratio for Black people from 0.33 (95 % CI 0.27 – 0.42) to 0.46 (0.39 – 0.55). The adjusted hazard ratios still showed significantly lower all-cause mortality in both South Asian and Black people compared to White people. The pattern of confounding was similar for both South Asian and Black people on risk of dying following self-harm in comparison with White people.

### Prevalence of risk factors for mortality

We examined the prevalence of socio-demographic factors, precipitating factors and clinical characteristics known to be associated with premature death [4, 5] in South Asian and Black people versus White people who presented to hospital for self-harm using chi square tests (Table 3). In comparison with White people, both South Asian and Black people were younger and less likely to be male. South Asian people who self-harmed were less likely to be unemployed than White people. Their occupation was more likely to be classified under household duties, whereas Black people who presented to hospital following self-harm were more likely than White people to be students. Both South Asian and Black people were more likely than White people to be living in the most deprived areas. South Asian people were less likely than White people to have physical health problems, and both South Asian and Black people were less likely than White people to have mental health problems identified at the time of the index self-harm episode. The South Asian and Black people who self-harmed were also less likely than White people to have consumed alcohol at index episode. South Asian and Black people both had lower prevalence of previous self-harm than White people, and were less likely to have had previous or current psychiatric treatment.

**Fig. 3:**
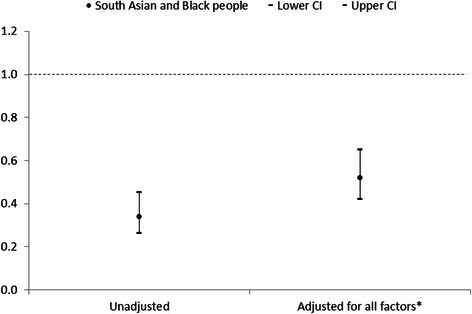
All-cause mortality hazard ratios for South Asian and Black people combined in comparison with White people adjusted for all measured risk factors*. *Aged over 35, male, unemployed, most deprived Index of Multiple Deprivation tertile, physical health problem, mental health problem, alcohol involved at attempt, any previous self-harm, current psychiatric treatment

Due to low numbers in both the South Asian and Black groups, we were unable to adjust the Cox proportional hazard ratio models for all measured risk factors for premature death. However, the hazard ratios for mortality for both South Asian and Black people are consistently lower than White people, and follow a similar pattern when adjusted for age, sex and deprivation tertile (Figs. 1 and 2). Therefore, we combined the South Asian and Black groups in order to compare all-cause mortality with White people, when adjusted for all measured risk factors for premature death (Fig. 3). Adjusting for all risk factors attenuated the group difference; the unadjusted hazard ratio for South Asian and Black people combined increased from 0.34 (95 % CI 0.26 – 0.45) to 0.52 (95 % CI 0.42 – 0.65), though they remained at a significantly lower risk of death following self-harm than White people.

## Discussion

### Summary of main findings

We found a lower risk of premature mortality in South Asian and Black people following self-harm than in White people. This was true for all-cause, internal and external causes of death, including suicide, and also following adjustment for age, sex, and deprivation tertile (IMD). When we adjusted for all measured risk factors for premature death, the group difference was attenuated somewhat but South Asian and Black people remained at substantially and significantly lower risk of death following self-harm than White people.

### Comparison with existing literature

Previous literature has reported that repetition of self-harm is less frequent in South Asian and Black people than in White people [7, 8], and here we report lower risk of mortality following self-harm in these groups than in White people. Previous analysis found that risk factors for premature death following self-harm were being male, being in an older age group, history of self-harm and history of or current psychiatric treatment [4, 5]. All of these risk factors for premature death were significantly lower in South Asian and Black people who self-harm in comparison with White people, a finding which is consistent with this observed difference in risk, and with findings from our previous study into ethnic differences in self-harm [7]. Although ethnic minority groups in the general population have a higher risk of disease compared with White people, for example for death from cardiovascular disease [10], their relative risk of mortality in the self-harm cohort compared with Whites is lower. This is perhaps because White people who self-harm are more socially deprived than the general population.

### Interpretation

UK ethnic minorities are over-represented in deprived inner-city areas [10]. Recent research has suggested that some ethnicity-related health inequalities are accounted for by socioeconomic status and area of residence [11]. The South Asian and Black people who died following self-harm had higher area-level deprivation scores, indicating greater deprivation than the White group. Research has shown that individual-level deprivation contributes more than area-level deprivation to the risk of repeat self-harm, and this may also be true for mortality risk following self-harm [19]. Our results indicate that South Asian and Black people are more likely to live in deprived areas than White people in this study cohort. Nonetheless, the White people are more likely to be deprived according to the individual-level indicator of deprivation: unemployment. This apparent paradox may be the key to explaining why cohort members from the two ethnic minority groups have a lower risk of dying prematurely. In terms of socioeconomic status (and related psychosocial risk factors), White cohort members are starkly different from the majority of people who live in their communities. They are much more deprived and are therefore likely to experience a higher degree of marginalisation and social exclusion. Furthermore our previous paper indicated that individual-level predictors are far stronger than area-level ones [19].

The lower prevalence of individual-level factors associated with premature death does not entirely account for South Asian and Black people having a lower risk of all-cause mortality following self-harm than White people. Despite higher deprivation scores, it may be that there are other unmeasured protective factors which contribute to the decreased risk of mortality in ethnic minority groups, such as social cohesion, religious beliefs, or different familial and community support systems, although recent research has suggested that social capital has no moderating effect between ethnic density and health outcomes, including mental health [20].

As discussed, our previous study found lower rates of repeat hospital presentation for self-harm in South Asian and Black people in comparison with White people [9], and this study has found lower risk of death following self-harm for these groups. It is possible that people within these groups who are at higher risk of mortality do not self-harm, or they may self-harm but be less likely to seek help. There is some support for this theory; an investigation of the Health Survey for England found that ethnic minority respondents were half as likely as White respondents to seek medical attention following self-harm, though the authors suggested that it was unclear whether this was due to a lack of access to services, or perceived stigma associated with suicidal behaviour [21].

### Strengths and limitations

To our knowledge, this is the first study to examine mortality following self-harm specifically in South Asian and Black people in the UK. This large cohort study used data collected from three English cities, with comprehensive follow-up data on mortality. Theoretically there could be potential bias if higher risk subgroups among South Asian and Black cohort members were more likely to emigrate and were thereby lost to follow-up. However, our retention figures for follow-up were in fact very high (above 99 %) and almost identical across the ethnic minority groups we examined. Though the overall sample size was large, numbers of those dying following ED presentation for self-harm were small, which somewhat limited the statistical power of the analysis. Ethnicity was known for 72 % of people presenting to hospital following self-harm, and is assigned by clinicians. There is potential bias in that information on ethnicity was not available for 28 % of people presenting to hospital following self-harm. It may be that the broad ethnic categories used in the analysis may help to alleviate some of this bias. We analysed broad ethnic groups due to small numbers, which does not account for increasing numbers of people from mixed heritage, and may not account for cultural identity [22]. However, this has allowed comparison with previous research on self-harm in ethnic minority groups in the UK. The overall low numbers in the ethnic minority groups means that detailed subgroup analysis by cause-specific mortality has not been possible. To achieve adequate power and multivariable model stability, it was necessary to combine South Asian and Black people to examine the contribution of all measured risk factors for premature death to the hazard ratios for death following self-harm. We acknowledge that these groups are not homogenous, although they show a similar pattern in hazard ratios for death following self-harm (Figs. 1 and 2), and have a similarly lower prevalence of risk factors for premature death following self-harm in comparison with White people. We have been unable to measure ethnic density, which may confer a buffer effect, whereby individuals from ethnic minority groups do better when living in an area with a high concentration of their own ethnicity [23–25]. This study is limited to data on people who present to hospital following self-harm. We cannot comment on any potential ethnic differences in self-harm rates and mortality in the community. It may be that the lower rates of self-harm and mortality in South Asian and Black people are due to reduced help-seeking behaviour following self-harm in these groups [21]. As the data is from five hospitals within three English cities of varying service provision and socioeconomic status, the findings should be generalizable more broadly to self-harm populations who present to emergency departments.

### Clinical implications and further research

We have shown that premature mortality following self-harm is low in South Asian and Black people in comparison with White people. On assessment clinicians may work with patients from these groups to help identify those factors that are protective, such as family support and social cohesion, as well as modifiable risk factors for poor outcome. Qualitative research methodology might help us further understand self-harming behaviour in UK ethnic minority communities, including episodes that never come to the attention of statutory medical services. It may be that identifying protective factors in this group can inform public health initiatives.

## Conclusion

This paper demonstrates that the risk of premature death following hospital presentation for self-harm is lower in South Asian and Black people than White people in the UK. South Asian and Black people are also less likely to have many of the risk factors known to be associated with premature death following self-harm. An awareness of both the risk and protective factors for premature death may help to inform clinical decisions as part of a comprehensive psychosocial assessment.
